# Supplementary material for: Inhibition of Tumor Growth by Dietary Indole-3-Carbinol in a Prostate Cancer Xenograft Model May Be Associated with Disrupted Gut Microbial Interactions
Source: Nutrients. 2019 Feb 22;11(2):467. doi: 10.3390/nu11020467 (PMC6413210; doi:10.3390/nu11020467)
Supplement: Supplementary file 1 [file nutrients-11-00467-s001.pdf]

Table S1. Microbial diversity of cecal samples in mice fed with or without in mice.

Diversity Indices	Control	Indol-3-carbinol	P value
**Chao1**	307±19.27	335.3±18.91	0.95
**PDwholetree**	15.64±0.61	16.92±0.5703	0.85
**Shannon**	4.133±0.1206	4.254±0.1332	0.79
**Simpson**	0.8782±0.01083	0.8869±0.01269	0.66

Table S2. The topological properties of microbial co-occurrence networks.

Group	Control (C)	I3C (I)
[a] No. of original OUTs	238	208
Similarity thresholds	0.80	0.79
[b] Network size	156	180
Avg. path	3.821	4.100
Avg. clustering coefficient	0.241	0.246
Modularity (No. of modules)	0.674 (15)	0.667 (15)

[a] The number of OTUs was originally used for network construction by the RMT-based network method. [b] The number of OTUs (i.e., nodes) in a network.

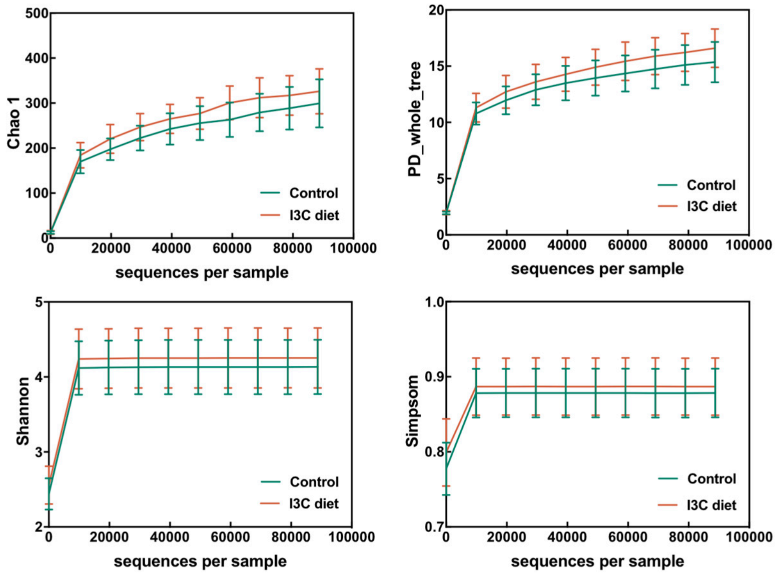

Figure S1. Rarefaction curves of cecal samples in mice fed with or without I3C.
